# The anti-ageing molecule sirt1 mediates beneficial effects of cardiac rehabilitation

**DOI:** 10.1186/s12979-017-0088-1

**Published:** 2017-03-16

**Authors:** Giusy Russomanno, Graziamaria Corbi, Valentina Manzo, Nicola Ferrara, Giuseppe Rengo, Annibale A. Puca, Salvatore Latte, Albino Carrizzo, Maria Consiglia Calabrese, Ramaroson Andriantsitohaina, Walter Filippelli, Carmine Vecchione, Amelia Filippelli, Valeria Conti

**Affiliations:** 1 Department of Medicine, Surgery and Dentistry, University of Salerno, Via S. Allende 43, 84081 Baronissi, Italy; 2 Department of Medicine and Health Sciences, University of Molise, Campobasso, Italy; 3 Department of Translational Medical Sciences, Federico II University of Naples, Naples, Italy; 4 Salvatore Maugeri Foundation, IRCCS, Scientific Institute of Telese Terme, Benevento, Italy; 5 IRCCS MultiMedica, Milan, Italy; 6 Cardiac Rehabilitation Unit of “San Gennaro dei Poveri” Hospital, Naples, Italy; 7 Vascular Physiopathology Unit, IRCCS INM Neuromed, Pozzilli, Italy; 8 INSERM U1063, Stress Oxydant et Pathologies Métaboliques, Institut de Biologie en Santé, Université d’Angers, Angers, France; 9 Department of Institutional Study and Territorial Systems, University of Naples “Parthenope”, Naples, Italy

**Keywords:** Heart failure, Rehabilitation, Sirtuin, Catalase, Oxidative stress, Endothelium

## Abstract

**Background:**

An exercise-based Cardiac Rehabilitation Programme (CRP) is established as adjuvant therapy in heart failure (HF), nevertheless it is underutilized, especially in the elderly. While the functional and hemodynamic effects of CRP are well known, its underlying molecular mechanisms have not been fully clarified. The present study aims to evaluate the effects of a well-structured 4-week CRP in patients with stable HF from a molecular point of view.

**Results:**

A prospective longitudinal observational study was conducted on patients consecutively admitted to cardiac rehabilitation. In fifty elderly HF patients with preserved ejection fraction (HFpEF), levels of sirtuin 1 (Sirt1) in peripheral blood mononuclear cells (PBMCs) and of its targets, the antioxidants catalase (Cat) and superoxide dismutase (SOD) in serum were measured before (Patients, P) and at the end of the CRP (Rehabilitated Patients, RP), showing a rise of their activities after rehabilitation.

Endothelial cells (ECs) were conditioned with serum from P and RP, and oxidative stress was induced using hydrogen peroxide. An increase of Sirt1 and Cat activity was detected in RP-conditioned ECs in both the absence and presence of oxidative stress, together with a decrease of senescence, an effect not observed during Sirt1 and Cat inhibition.

**Conclusions:**

In addition to the improvement in functional and hemodynamic parameters, a supervised exercise-based CRP increases Sirt1 activity and stimulates a systemic antioxidant defence in elderly HFpEF patients. Moreover, CRP produces antioxidant and anti-senescent effects in human endothelial cells mediated, at least in part, by Sirt1 and its target Cat.

## Background

Despite recent advances in clinical/diagnostic tools and therapies, the incidence and prevalence of Heart Failure (HF) show a steady increase [1]. A cardiac rehabilitation programme (CRP) based on exercise training, has been recognized as a fundamental component in the continuum of care for patients with HF. Meta-analyses of randomized controlled trials on CRPs have demonstrated a significant reduction of all-cause mortality, with lower rates of re-infarction and cardiac mortality [2].

However, these studies included very few elderly or high-risk patients, and exercise is rarely viewed as a necessary prescription for these patients because they have more barriers to participation in exercise training [3].

In HF patients, exercise was shown to be associated with significant improvement in functional and hemodynamic parameters [4–7], nevertheless there are few data explaining the molecular mechanisms underlying exercise-based CRP.

It has been established both in humans and in animal models that exercise training can stimulate the natural antioxidant defences thereby contrasting reactive oxygen species (ROS) accumulation [8, 9].

The NAD^+^-dependent deacetylase sirtuin 1 (Sirt1) is now recognized as a mediator of the response to oxidative stress and endothelial dysfunction, phenomena both correlated with endothelial cell pathophysiology and Cardiovascular Diseases (CVDs), including HF [10]. Evidence about the protective role of Sirt1 in vascular biology has indicated Sirt1 as a possible target in preventing CVDs and other diseases [11, 12]. Indeed, Sirt1 plays a crucial role in both cellular senescence and ageing, and it was recognized as modulator of the oxidative stress response by inducing the expression of antioxidant enzymes such as superoxide dismutase (SOD) and catalase (Cat) [13, 14]. Recently, Lu et al. [15] showed that in advanced HF, low Sirt1 expression in ageing might be a significant contributing factor in the downregulation of antioxidants and upregulation of oxidative stress and apoptosis. We previously showed that moderate exercise promoted Sirt1 activity in rats and induced increasing SOD and Cat expression. Notably, in aged sedentary rats there was lower levels of Cat comparing to young rats and exercise led to a complete recover of such antioxidant enzyme [16].

Cat, a molecular target of Sirt1, represents a primary safeguard of the antioxidant system [17], and recent studies have suggested that this enzyme might play an important role in the pathophysiology of HF [18, 19].

In humans, exercise training improves cardiovascular function and endothelial homeostasis, although the benefit achieved varies widely depending on the type and duration of exercise [20, 21].

In the present study, we aim at investigating the molecular changes possibly induced by a 4-week CRP on Sirt1 activity in peripheral blood mononuclear cells (PBMCs) and antioxidant status in serum of patients with stable HF. Moreover, we looked at changes induced by the conditioning of human endothelial cells, exposed or not to oxidative stress induced by H_2_O_2_, with serum isolated from patients before and at the end of the CRP.

## Results

### Heart failure elderly patient recruitment and characterisation

Fifty-three consecutive patients affected by HF were recruited from the Cardiac Rehabilitation Unit. All patients completed the CRP. As only three patients were women, they were excluded from the analysis. Therefore, the final study population consisted of 50 elderly male patients (mean age 68.6 ± 6.3 years). None of the patients had experienced a myocardial infarction (MI) in the 12 months preceding the study. All patients were in clinically stable condition, and classified as in NYHA II and III class with a preserved Ejection Fraction (EF) [10 patients with HF mid-range EF; 40 with HF preserved EF]. All definitions were based on the ESC and ACCF/AHA criteria, in which the term “stable” defines treated patients with symptoms and signs that have remained generally unchanged for at least 1 month [22].

The clinical and demographic features of the study population are listed in Table 1. Information on comorbidities and concomitant medications were gathered from all patients. No racial/ethnic-based differences were present. At baseline, no differences in medical therapy were found, and no changes occurred during the study period.

**Table 1:** Study population characteristics and medication use

Age (y.o.), *mean (SD)*	68.6 (6.3)	Medications, *n (%)*	
Gender *(M/F)*	50/0	β-blockers	46 (92)
BMI (kg/m^2^), *mean (SD)*	28.03 (3.17)	ACE-inhibitors	29 (58)
SBP (mmHg), *mean (SD)*	122 (6)	ARBs	9 (18)
DBP (mmHg), *mean (SD)*	80 (9)	Diuretics	8 (16)
HR (bpm), *mean (SD)*	84 (8)	Nitrates	6 (12)
CAD, *n (%)*	49 (98)	Ca^2+^-antagonists	4 (8)
ischemic	47 (94)	α-antagonists	2 (4)
hypertrophic	1 (2)	Aspirin	46 (92)
dilatative	1 (2)	Anticoagulants	31 (62)
PTCA, *n (%)*	33 (66)	Other cardiac drugs	3 (6)
CABG, *n (%*)	9 (18)	Antiarrhythmics	2 (4)
Valvular substitution, *n (%*)	1 (2)	Statins	49 (98)
Smoking, *n* (*%*)	35 (70)	Gastro-protective drugs	44 (88)
Familiarity, *n* (*%*)	15 (30)	Polyunsaturated fats	13 (26)
Hypertension, *n* (*%*)	29 (58)	Oral hypoglycaemics	9 (18)
Dyslipidaemia, *n* (*%*)	28 (56)	Insulin	5 (10)
Diabetes, *n* (*%*)	13 (26)		
COPD, *n* (*%*)	6 (12)		
Obesity, *n* (*%*)	4 (8)		
Peripheral Artery Disease, *n* (*%*)	2 (4)		
Arrhythmias, *n* (*%*)	2 (4)		
Distyroidism, *n* (*%*)	1 (2)		
Other diseases, *n* (*%*)	1 (2)		

Data are expressed as mean (SD) or number of subjects (%). BMI, Body Mass Index; SBP, Systolic Blood Pressure; DBP, Diastolic Blood Pressure; HR, Heart Rate; bpm, beat/minutes; CAD, Coronary Artery Disease; PTCA, Percutaneous Transluminal Coronary Angioplasty; CABG, Coronary Artery Bypass Graft; COPD, Chronic Obstructive Pulmonary Disease; ARBs, Angiotensin II Receptor Blockers.

Biochemical, echocardiographic and cardiopulmonary stress test features of patients before (P) and at the end of the CRP (RP) are shown in Table 2. A CRP significantly reduced cholesterol and increased creatinine levels (both *P* < 0.0001).

**Table 2:** Changes in biochemical, echocardiographic and cardiopulmonary stress test parameters induced by CRP

	P	RP	*P* value
Biochemistry			
Cholesterol (mmol/L)	157.61 ± 37.11	150.67 ± 30.37	*<0.0001*
Creatininemia (mmol/L)	0.94 ± 0.24	0.97 ± 0.20	*<0.0001*
Hemoglobin (g/dL)	13.58 ± 1.35	13.42 ± 0.53	0.494
Echocardiographic parameters			
EF (%)	53.33 ± 8.97	55.10 ± 6.79	*0.011*
LVEDD (mm)	51.37 ± 3.77	51.31 ± 3.13	0.804
Cardiopulmonary stress test			
SBP max (mmHg)	169.02 ± 15.54	165.51 ± 19.29	*0.002*
DBP max (mmHg)	81.46 ± 5.62	80.64 ± 5.15	0.077
HR max (bpm)	117.56 ± 21.55	123.13 ± 13.20	*0.034*
Rate-pressure product (mmHg x bpm)	19892.69 ± 3841.64	20261.54 ± 3743.32	*<0.0001*
Test duration (sec)	359.41 ± 112.57	451.89 ± 109.78	*<0.0001*
VO2 max (ml/kg/min)	20.30 ± 4.61	24.51 ± 5.81	*<0.0001*

P, HF patients pre-CRP; RP, the same patients post-CRP. Data are expressed as mean ± SD. *EF*, Ejection Fraction; *LVEDD*, Left Ventricle End Diastolic Diameter; *SBP*, Systolic Blood Pressure; *DBP*, Diastolic Blood Pressure; *HR*, Heart Rate; bpm, beat/minutes. A *P* value <0.05 was considered significant

Cardiopulmonary stress test revealed a reduction in maximum systolic blood pressure (*P* = 0.002), and increased maximum heart rate (*P* = 0.034), rate-pressure product (*P* < 0.0001), test duration (*P* < 0.0001), and VO2 max (*P* < 0.0001) with consequent significantly higher exercise tolerance, one of the most crucial target in the HF treatment, after CRP.

### CRP-induced changes in antioxidant capacity in heart failure elderly patients

The activity of Sirt1 and of its molecular targets, Cat and SOD before and at the end of the CRP were evaluated.

**Fig. 1:**
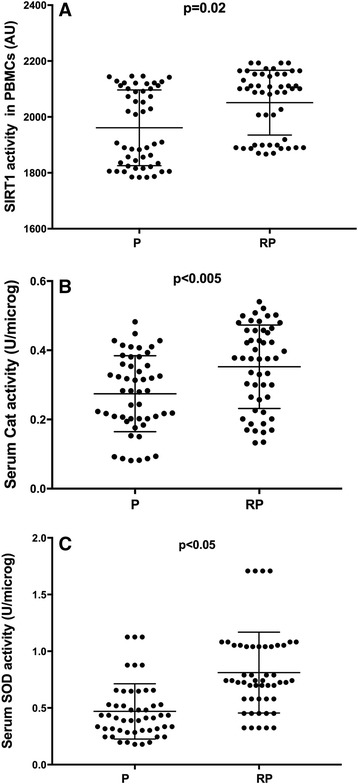
CRP increased Sirt1 activity in PBMCs with a concomitant rise of antioxidants in sera. Sirtuin 1 (Sirt1) activity **(a)** was determined in the nuclei extracted from PBMCs from patients before (P) and after 4 weeks of a cardiac rehabilitation programme (CRP). Catalase (Cat) activity **(b)** and superoxide dismutase (SOD) activity **(c)** were determined in serum samples

The CRP enhanced Sirt1 activity measured in PBMCs from patients (RP *vs* P, *P* = 0.02) (Fig. 1, Panel a). Likewise, Cat and SOD activities measured in serum were greater in RP than in P (*P* < 0.005 and *P* < 0.05, respectively) (Fig. 1, Panels b and c).

### Sirt1, Cat and SOD activities in endothelial cells conditioned with patients’ sera

To investigate the possible role of Sirt1, Cat and SOD in modulating the beneficial effects of the CRP, an *in vivo-in vitro* model was set up by conditioning human endothelial cells (ECs) with sera from patients at time 0 (Patient serum-conditioned ECs, P-ECs) and at the end of the CRP (Rehabilitated Patient serum-conditioned ECs, RP-ECs). Moreover, the antioxidant response in such conditioned cells was evaluated after the induction of stress using H_2_O_2_.

**Fig. 2:**
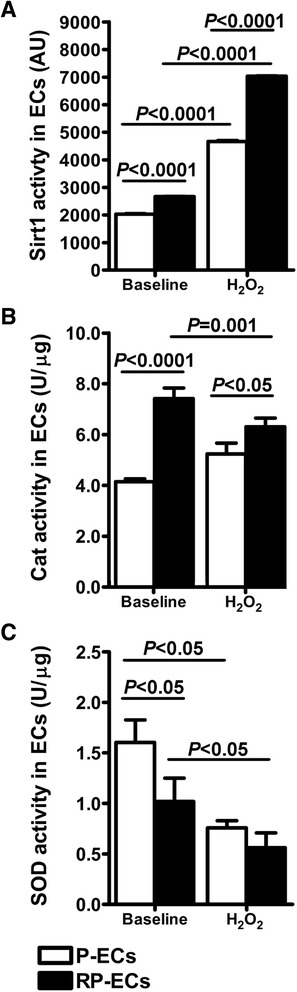
Sirt1, Cat and SOD activities in conditioned endothelial cells. Sirt1 **(a)**, Cat **(b)** and SOD **(c)** activities in endothelial cells (ECs) conditioned with sera from patients before (P) and after (RP) a 4-week well-structured CRP in either the presence or absence of H_2_O_2_-induced oxidative stress

Sirt1 and Cat activities were higher in RP-ECs than in P-ECs (both, *P* < 0.0001) (Fig. 2, Panels a and b). Conversely, SOD activity decreased in RP-ECs (*P* < 0.05) compared with P-ECs (Fig. 2, Panel c).

In the presence of H_2_O_2_-induced oxidative stress, Sirt1 and Cat activities were higher in RP-ECs than in P-ECs (*P* < 0.0001 and *P* < 0.05, respectively) (Fig. 2, Panels a and b), whereas SOD activity did not change (Fig. 2, Panel c).

These results showed that a CRP induced Sirt1 and Cat activation in both the absence and presence of oxidative stress, suggesting the role of Sirt1 in stimulating the antioxidant response.

### Role of Sirt1 and Cat in endothelial cell senescence

**Fig. 3:**
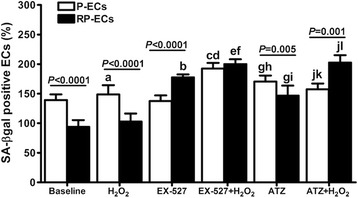
Effect of the inhibition of Sirt1 and Cat activities on endothelial cell senescence. SA-β-gal staining of ECs conditioned with sera from HF patients in the presence and absence of oxidative stress. Sirt1 and Cat activities were inhibited by EX-527 and 3-amino-1,2,3-triazole (ATZ), respectively. Senescence values are shown as a percentage of the reference condition (FBS-conditioned ECs), which is 100%. a) *P* < 0.05 *vs* baseline; b) *P* = 0.001 *vs* baseline; c) *P* < 0.05 *vs* H_2_O_2_; d) *P* = 0.01 *vs* EX-527; e) *P* = 0.001 *vs* H_2_O_2_; f) *P* < 0.02 *vs* EX-527; g) *P* < 0.0001 *vs* baseline; h) *P* < 0.01 *vs* EX-527;i) *P* < 0.05 *vs* EX-527; j) *P* < 0.05 *vs* ATZ; k) *P* < 0.02 *vs* EX-527 + H_2_O_2_; l) *P* < 0.0001 *vs* H_2_O_2_. (B): a) *P* = 0.001 *vs* baseline; b) *P* < 0.02 *vs* baseline; c) *P* < 0.005 *vs* baseline; d) *P* = 0.002 *vs* H_2_O_2_; e) *P* = 0.01 *vs* EX-527; f) *P* < 0.01 *vs* baseline; g) *P* < 0.05 *vs* EX-527; h) *P* < 0.05 *vs* H_2_O_2_; i) *P* = 0.002 *vs* EX-527 + H_2_O_2_; j) *P* = 0.001 *vs* EX-527 + H_2_O_2_; k) *P* < 0.005 *vs* H_2_O_2_

The senescence in endothelial cells (ECs) conditioned with patients’ sera was measured. ECs conditioned with sera from patients at the end of a 4-week CRP (RP-ECs) showed a significantly reduced senescence compared to that conditioned with sera from patients before CRP (P-ECs), in both the absence and presence of induced oxidative stress (both, *P* < 0.0001; Fig. 3).

To investigate the possible role played by Sirt1 and its molecular target Cat in the modulation of cell senescence, P-ECs and RP-ECs, either exposed or not to oxidative stress, were treated with Sirt1 and Cat pharmacological inhibitors, EX-527 and 3-amino-1,2,4-triazole (ATZ) respectively.

As shown in Fig. 3, the inhibition of Sirt1 activity by EX-527 caused an increase of senescence in RP-ECs compared with baseline (*P* = 0.001), but not in P-ECs. Interestingly, in the presence of H_2_O_2_ oxidative stress, EX-527 induced a rise in senescence, in both P-ECs (*P* < 0.05) and RP-ECs (*P* = 0.001). Hence, Sirt1 inhibition abolished the anti-senescent effect of a CRP, suggesting Sirt1 as a modulator of endothelial cell senescence.

Also the inhibition of Cat activity by ATZ resulted in an increased senescence, in both P-ECs and RP-ECs (both, *P* < 0.0001), compared with baseline. In the presence of H_2_O_2_-induced oxidative stress, ATZ treatment caused a significant increase in the senescence rate in ECs conditioned with RP sera (*P* < 0.0001) compared with basal levels. Of note, senescence become higher in stressed RP-ECs compared with stressed P-ECs when Cat was inhibited (*P* = 0.001). These data suggest that Cat is, at least in part, responsible for the reduced senescence rate observed in ECs conditioned with RP sera.

## Discussion

Most of the studies in both young and older adults have been planned after considering functional and hemodynamic outcomes, without clarifying the effects of a CRP from a molecular point of view.

In this study, we showed an increase of Sirt1 activity in PBMCs alongside an increase of antioxidant capability in serum isolated from patients with HF after 4 weeks of a CRP.

*In vivo-in vitro* experiments performed in endothelial cells conditioned with patients before and after CRP showed that serum from the rehabilitated patients is able to stimulate Sirt1 activity and the cellular antioxidant defence by increasing activity of the Sirt1 target Cat.

Furthermore, the conditioning of human endothelial cells with serum from rehabilitated patients attenuated senescence in both the absence and presence of oxidative stress induction and such effect was eliminated by the pharmacological inhibition of Sirt1 or Cat activity.

Cellular senescence is a hallmark of ageing and a process in which competent cells are brought into a permanent form of growth arrest. If and how senescence is correlated with age-associated frailty and diseases is still one of the major unanswered questions in ageing physiology and clinical geriatrics [23]. An increase of oxidative stress-induced senescence can be dangerous to endothelial cells, resulting in impairment of endothelial structure and function. Some authors showed that cellular senescence is involved in endothelial dysfunction and atherogenesis, and this was confirmed by a histological study on atherosclerotic human plaques demonstrating morphological features of senescence [24]. As oxidative stress-induced endothelial dysfunction is strictly connected to HF, researching methods to modify this condition is certainly of clinical interest.

The role played by Sirt1 in the regulation of ageing, endothelial homeostasis and cellular senescence is now recognized. Indeed, several studies demonstrated that a H_2_O_2_ treatment caused a reduction of Sirt1 protein expression, and the inhibition of Sirt1 contributed to a H_2_O_2_-induced senescence in endothelial cells [25, 26]. Furthermore, the Sirt1 target Cat was also shown to be involved in ageing and senescence control [27, 28]. We previously demonstrated that Cat is reduced during ageing [16], and involved in the reduction of endothelial senescence during an aerobic exercise training [20, 21]. Some studies in animal models demonstrated that over-expression of Cat in heart and vessels may have a beneficial impact on HF. In particular, Cat may prevent adverse myocardial remodelling and contribute to the preservation of geometric and functional changes by alleviating stress in the endoplasmic reticulum [18, 29].

Notably, patients enrolled in the present study were HF elderly patients with preserved ejection fraction, a phenotype of HF that is attracting particular attention from both physicians and researchers. Actually, pharmacological trials performed to assess improving of outcome and symptoms, including exercise intolerance, in HFpEF patients have been shed light the absence of effective drugs [30, 31].

On the other hand, some recent studies in such patients have suggested that exercise training is a promising therapeutic strategy to improve exercise intolerance [32], increase exercise capacity, as measured objectively using peak oxygen consumption, and ameliorate quality of life and diastolic function, assessed by echocardiography [6, 7, 33].

Here we showed that, in addition to the improvement of hemodynamic parameters and exercise tolerance (assessed by cardiopulmonary stress test), an exercise-based CRP increases Sirt1 activity and stimulates a systemic antioxidant defence in HFpEF elderly patients and was able to produce antioxidant and anti-senescent effects in endothelial cells mediated, at least in part, by Sirt1 and its target Cat.

### Limitations

A possible limitation of the present study could be the lack of a group of heart failing patients not undergone CR. However, the main outcome was represented by the investigation of the molecular changes occurred before and after a well-structured 4-week rehabilitation program in patients affected by chronic HF, who did not change their clinical characteristics and pharmacological therapy during the study period.

Another drawback is the lack of women in the study population. Actually, we did recruit only three women, and then we decided to exclude them because of the small number. This is in line with the fact that women are less inclined to take part in cardiac rehabilitation programs [34]. Therefore, further studies are necessary to better clarify the molecular effects of CR also in female patients.

## Conclusion

The ability of exercise training to regulate vascular endothelial function and oxidative stress response is an example of how lifestyle and/or tools such as exercise-based CRPs can complement both clinical and pharmacological means of managing CVDs. In particular, cardiac rehabilitation is a helpful medical practice in which several molecular factors mutually influence each other. The exercise training included in CRPs acts as a non-pharmacological inductor of antioxidant response.

To determine the molecular mechanisms underlying the beneficial effects of CRPs is an essential step in developing a strategy to facilitate the clinical practice of exercise training. Further studies should be addressed to evaluate the possibility of reducing the number and the dosage of drugs in HF patients, including those with preserved ejection fraction by implementing exercise programs, especially in high-risk elderly subjects.

## Methods

### Study Design and Population

A prospective longitudinal observational study was conducted on patients consecutively admitted to the Cardiac Rehabilitation Unit of “San Gennaro dei Poveri” Hospital in Naples, Italy. Patients’ information and consent forms were approved by the ethical committee of ASL of Salerno Registry of Observational Studies (RSO) n.10/14.

The study was performed in accordance with the Declaration of Helsinki Seventh Revision (2013) and its amendments. This report adheres to the standards for the reporting of observational trials and was written according to the STROBE guidelines for Observational Studies in Epidemiology - Molecular Epidemiology (STROBE-ME) [35].

Exclusion criteria included unstable angina pectoris, uncompensated HF, complex ventricular arrhythmias, pacemaker implantation and orthopaedic or neurological limitations to exercise.

All enrolled patients underwent a physical examination, collection of demographic and routine blood chemistry tests, chest X-ray, blood pressure measurement, electrocardiographic and echocardiographic examinations, cardiopulmonary stress test and a 6-min walking test with Borg index evaluation. For interval training, low muscle commitment calisthenics and respiratory exercises were performed.

### Training Protocol

The CRP consisted of 30-min sessions of aerobic exercise, 5 days a week. A daily training session comprised a warm-up (10 min), endurance training (15 min) and a cool-down (5 min) on a cycle ergometer at 50% of the VO2 max achieved on the cardiopulmonary stress test.

### Blood Sample Collection

Overnight fasting blood samples were obtained from patients before starting and at the end of the CRP. After centrifugation at 1500 × *g* for 10 min, serum samples were transferred to new tubes and stored at −80 °C until analysis. PBMCs were isolated from whole blood by Ficoll-Paque PLUS (GE Healthcare, Munich, Germany), according to manufacturer’s procedures.

Samples isolated from patients before CR were indicated as P, while those collected from patients after CR were designated as RP.

### Cell Culture and Treatments

Human Umbilical Vein Endothelial Cells (HUVECs, ECs) were purchased from Clonetics (Walkersville, MD). The cells were cultured in an endothelial growth medium, containing FBS at a concentration of 2% and bovine brain extract (with FGF-2 at a concentration of 100–500 pg/ml). The cells were subcultured by trypsinization, seeded on cell culture dishes coated with 0.1% gelatin and growth in an atmosphere of 5% CO2 at 37 °C. Pilot experiments to identify the concentration of hydrogen peroxide (H_2_O_2_ = 100–750 μM) that effectively induced a significant decrease in the survival of control cells, were conducted and a concentration of 500 μM was chosen. Moreover, we evaluated the effect of oxidative stress 12, 24, 48, and 72 h after a treatment with 500 μM H_2_O_2_. Finally, we chose the time of 48 h as representative of the most relevant change.

Therefore, ECs were seeded and cultured for 48 h in a medium supplemented with either the patient’s serum (10%) at time 0 (Patient serum-conditioned ECs, P-ECs) and post CR (Rehabilitated Patient serum-conditioned ECs, RP-ECs), or FBS (10%) as a control and were exposed or not to oxidative stress induced by 500 μM H_2_O_2_. Four hours after H_2_O_2_ exposure, the growth medium was replaced with fresh medium containing FBS.

All experiments were performed at a population doubling level (PDL) of 8 to 12.

### Sirt1 Activity

Crude nuclear samples were extracted by suspending the cells into 1 mL of lysis buffer (10 mM of Tris HCl at pH 7.5, 10 mM of NaCl, 15 mM of MgCl_2_, 250 mM of sucrose, 0.5% NP-40, 0.1 mM of EGTA). Cells were spun through 4 ml of sucrose cushion (30% sucrose, 10 mM of Tris HCl at pH 7.5, 10 mM of NaCl, 3 mM of MgCl_2_) at 1300 × *g* for 10 min at 4 °C. The isolated nuclei were suspended in 50–100 μl of extraction buffer (50 mM of HEPES KOH at pH 7.5, 420 mM of NaCl, 0.5 mM of EDTA Na_2_, 0.1 mM of EGTA, 10% glycerol). After centrifugation at 15,000 rpm for 10 min, the protein concentration of the crude nuclear extract without protease inhibitor was determined by the Bradford method. Sirt1 activity in the nuclei was determined using the CycLex SIRT1/Sir2 Deacetylase Fluorometric Assay Kit (Ina, Nagano, Japan). The reaction was carried out by simultaneously mixing fluorescent-labeled acetylated peptide as substrate and 10 μl of the sample, trichostatin A, NAD, and lysyl endopeptidase. The intensity of the fluorescence at 440 nm was measured 60 min after the onset of the reaction. Values are reported as relative fluorescence/μg of protein (AU). All data are the means ± standard deviation (SD) of three independent experiments.

### Catalase and Superoxide Dismutase Antioxidant Activities

Catalase (Cat) and superoxide dismutase (SOD) activities were determined using the Catalase Assay Kit and the Superoxide Dismutase Assay Kit (Cayman Chemical, USA). Samples were previously diluted with buffer (1:10 for serum; 1:2 for cell lysate). The values were reported as U/μg of protein. All data are the means ± SD of three independent experiments.

### Sirt1 and Catalase Activities Inhibition

To investigate if Sirt1 or Cat activity influenced changes in the senescence of the conditioned cells either exposed or not to H_2_O_2_, their respective activities were inhibited using EX-527 (Sigma, Milan-Italy) at a concentration of 5 μM for 1 h and 3-amino-1,2,4-triazole (ATZ) (Sigma, Milan-Italy) at a concentration of 10 mM for 3 h.

### Senescence-Associated β-galactosidase (SA-β-gal) Activity

Cultured cells were washed in PBS and fixed with 2% formaldehyde and 2% glutaraldehyde for 10 min at room temperature. The cells were washed and then incubated at 37 °C in staining buffer with the following components: 40 mM citric acid/sodium phosphate (pH 6.0), 0.15 M NaCl, 2 mM MgCl_2_, 5 mM potassium ferrocyanide, and 1 mg/mL X-gal (5-bromo-4 chloro-3-indolyl β-D-galactoside). After 4 h, the SA-β-gal rate was obtained by counting four random fields per dish and assessing the percentage of SA-β-gal-positive cells from 100 cells per field. Senescence values are shown as a percentage of the reference condition (FBS-conditioned ECs), which is 100%.

### Statistical Analysis

Continuous variables are expressed as mean ± SD and compared by paired or unpaired Student’s *t* test (normally distributed variables) or by two or three way ANOVA when appropriate, or as median ± interquartile range value and compared by the Mann–Whitney *U* test (not normally distributed). Normality of data distribution was evaluated using the Kolmogorov-Smirnov test. Non-normally distributed continuous variables were converted to their natural log functions. Categorical variables are expressed as a proportion and compared by the *χ* ^2^ test, with risk ratios and 95% confidence intervals quoted.

All data were analysed using SPSS version 19.0 (SPSS, Inc., Chicago, Illinois-USA). Statistical significance was accepted at *P <* 0.05.

## Abbreviations

EX-527: Sirtuin 1 inhibitor
ACCF: American College of Cardiology Foundation
AHA: American Heart Association
ARBs: Angiotensin II Receptor Blockers
ATZ: 3-amino-1,2,4-triazole
AU: Arbitrary unit
BMI: Body mass index
bpm: Beat/minutes
CABG: Coronary artery bypass graft
CAD: Coronary artery disease
Cat: Catalase
COPD: Chronic obstructive pulmonary disease
CRP: Cardiac rehabilitation programme
CVDs: Cardiovascular diseases
DBP: Diastolic blood pressure
ECs: Endothelial cells
EF: Ejection fraction
EGTA: Ethylene glycol tetraacetic acid
ESC: European Society of Cardiology
FBS: Foetal bovine serum
H_2_O_2_: Oxygen peroxide
HCl: Hydrogen chloride
HEPES: 4-(2-hydroxyethyl)-1-piperazineethanesulfonic acid
HF: Heart failure
HR: Heart rate
KOH: Potassium hydroxide
LVEDD: Left ventricle end diastolic diameter
MgCl_2_: Magnesium chloride
MI: Myocardial infarction
MnSOD: Manganese-dependent superoxide dismutase
NaCl: Sodium chloride
NAD^+^: Nicotinamide adenine dinucleotide coenzyme
NP-40: 4-nonylphenyl-polyethylene glycol
NYHA: New York Heart Association functional classification of heart failure
O_2_: Oxygen
P: Patients before cardiac rehabilitation programme
PDL: Population doubling level
P-ECs: Patient serum-conditioned endothelial cells
PTCA: Percutaneous transluminal coronary angioplasty
ROS: Reactive oxygen species
RP: Rehabilitated patients
RP-ECs: Rehabilitated patient serum-conditioned endothelial cells
SA-β-gal: Senescence-Associated β-galactosidase
SBP: Systolic blood pressure
Sirt1: Sirtuin 1
SOD: Superoxide dismutase
VO2: Maximal oxygen consumption
X-gal: 5-bromo-4 chloro-3-indolyl β-D-galactoside.
